# Small-angle X-ray scattering in the era of fourth-generation light sources

**DOI:** 10.1107/S1600576723004971

**Published:** 2023-06-23

**Authors:** Theyencheri Narayanan, William Chèvremont, Thomas Zinn

**Affiliations:** a ESRF – The European Synchrotron, 38043 Grenoble, France; b Diamond Light Source, Didcot OX11 0DE, United Kingdom; Brazilian Synchrotron Light Laboratory, Brazil

**Keywords:** small-angle X-ray scattering, ultra-small-angle X-ray scattering, X-ray photon correlation spectroscopy, fourth-generation synchrotrons, active colloids, soft matter systems, radiation damage

## Abstract

Recently, fourth-generation synchrotron sources with several orders of magnitude higher brightness and higher degree of coherence compared with third-generation sources have come into operation. These new X-ray sources offer exciting opportunities for the investigation of soft matter and biological specimens by small-angle X-ray scattering (SAXS) and related scattering methods. The improved beam properties together with the advanced pixel array detectors readily enhance the angular resolution of SAXS and ultra-small-angle X-ray scattering in the pinhole collimation. The high degree of coherence is a major boost for the X-ray photon correlation spectroscopy (XPCS) technique, enabling the equilibrium dynamics to be probed over broader time and length scales. This article presents some representative examples illustrating the performance of SAXS and XPCS with the Extremely Brilliant Source at the European Synchrotron Radiation Facility. The rapid onset of radiation damage is a significant challenge with the vast majority of samples, and appropriate protocols need to be adopted for circumventing this problem.

## Abstract 2

This article presents some representative examples illustrating the performance of small-angle X-ray scattering and X-ray photon correlation spectroscopy methods with the Extremely Brilliant Source at the European Synchrotron Radiation Facility.

## 1. Introduction

Over the past three decades, third-generation synchrotron sources have enabled significant broadening of the scope of small-angle X-ray scattering (SAXS) and related methods in the investigation of soft matter and biophysical systems (Narayanan & Konovalov, 2020 ▸; Jeffries *et al.*, 2021 ▸). The high brilliance of these sources facilitated time-resolved experiments in the millisecond range, even with low-contrast samples, and high angular resolution and spatially resolved measurements (Narayanan & Konovalov, 2020 ▸). Parallel developments of advanced detectors, sample environments and, most importantly, new data analysis methods were pivotal in exploiting the source properties (Jeffries *et al.*, 2021 ▸). As a result, SAXS methods allow simultaneous access to a broad range of size and time scales, deciphering the structural information from sub-nanometre to micrometre size scales and kinetics down to the sub-millisecond time range in hierarchically organized systems. Applications range from soft matter self-assembly to cellular processes under thermodynamically and physiologically relevant conditions (Narayanan & Konovalov, 2020 ▸; Ma & Irving, 2022 ▸).

The fourth-generation synchrotron sources based on multi-bend achromat storage-ring lattices (Eriksson *et al.*, 2014 ▸; Raimondi *et al.*, 2021 ▸; Liu *et al.*, 2022 ▸) are even more attractive for performing scattering experiments. Compared with the third-generation storage rings, these new sources have increased the brilliance and the degree of transverse coherence of X-ray beams by more than an order of magnitude. The enhanced brightness and coherence are very beneficial for SAXS and X-ray photon correlation spectroscopy (XPCS). Usually, SAXS measurements are performed using a larger beam consisting of multiple coherent volumes while XPCS requires a single (or a few) coherent scattering volume(s). For SAXS experiments, the key advantage of the new sources is the higher angular resolution due to the smaller beam cross section and divergence in the horizontal direction. These properties in turn allow relaxation of the collimation and reduction of the parasitic background at ultra-small angles (Narayanan *et al.*, 2022 ▸). The higher degree of coherence enables multispeckle XPCS measurements on dilute suspensions (Zinn *et al.*, 2022 ▸) or weakly scattering concentrated samples (Chushkin *et al.*, 2022 ▸) and probes the equilibrium dynamics down to the microsecond range. In addition, the development of high frame rate pixel array detectors is indispensable for exploiting the coherence properties in XPCS (Zhang *et al.*, 2018 ▸; Zinn *et al.*, 2018 ▸; Lehmkühler *et al.*, 2021 ▸).

This article presents some representative examples of SAXS and XPCS performed using the Extremely Brilliant Source (EBS) at the European Synchrotron Radiation Facility (ESRF) (Raimondi *et al.*, 2021 ▸). The EBS is based on the hybrid multi-bend achromat design and operates at 6.0 GeV electron energy with operational root-mean-square horizontal and vertical emittances of 130 and 10 pm rad, respectively (Raimondi *et al.*, 2021 ▸). The experiments reported here were performed at beamline ID02, which is a multipurpose X-ray scattering instrument covering a broad range of scattering vector magnitudes from 0.001 to 50 nm^−1^ with time resolution down to the submillisecond range (Narayanan *et al.*, 2022 ▸). By selecting a coherent beam, XPCS is usually performed in the ultra-small-angle X-ray scattering (USAXS) configuration.

## 2. Experimental methods

The main components of the ID02 time-resolved USAXS (TRUSAXS) beamline are the undulator source, the cryogenically cooled monochromator, focusing mirror optics, collimation slits, sample environments and the detector tube that houses different detectors (Narayanan *et al.*, 2018 ▸). The required collimation is obtained by three well separated slits in combination with the mirror focusing, and the last slit curtails the parasitic background in the ultra-small-angle region. For XPCS, an additional slit is used to select a single (or near single) coherent patch in the beam. The main technical feature of the TRUSAXS instrument is the evacuated detector tube, which is 34 m in length and 2 m in diameter. The SAXS/USAXS/XPCS detectors are enclosed within a wagon inside the detector tube that travels along a rail system from about 1 to 31 m. Optionally, a wide-angle X-ray scattering (WAXS) detector is placed outside the detector tube. For standard SAXS/WAXS/USAXS measurements, a scattering-vector range of 0.002 ≤ *q* ≤ 50 nm^−1^ is covered by these two sample-to-detector distances (1 and 31 m) with a single beam setting for an X-ray wavelength of 1 Å [*q* is the magnitude of the scattering vector given by 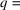 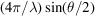 and θ is the scattering angle].

In the high-resolution configuration, 2D USAXS patterns down to *q* < 0.001 nm^−1^ can be recorded, or multispeckle ultra-small-angle XPCS (UA-XPCS) measurements can be performed with sufficiently scattering samples (Narayanan *et al.*, 2022 ▸). The EBS enables relaxation of the collimation conditions and maintains a lower parasitic background for the high-brilliance (2–6 × 10^13^ photons s^−1^ at 12.23 keV) operation. The high-resolution mode requires a tighter collimation but still provides a flux in excess of 10^12^ photons s^−1^. The coherent beam is defined by closing the primary slits (at 27 m from the source) to 0.15 mm × 0.15 mm and the last two secondary slits (at 49 and 62 m from the source) to 0.04 mm × 0.015 mm along the vertical and horizontal directions, respectively. The resulting beam is roughly symmetrical, with full width at half-maximum (FWHM) size ≃ 25 µm at the sample position. The SAXS/USAXS measurements are carried out using an Eiger2 4M (Dectris) hybrid pixel array detector, and for XPCS the PSI version of an Eiger 500 K is used (Narayanan *et al.*, 2022 ▸; Zinn *et al.*, 2018 ▸). The maximum frame rates of these detectors are 1000 and 23 000 s^−1^, respectively. Measured 2D SAXS/USAXS patterns are normalized and azimuthally averaged via the online data-reduction pipeline *Dahu* (Kieffer & Drnec, 2021 ▸). The 1D scattering profiles are further treated using the *SAXSutilities2* software (Sztucki, 2021 ▸). For XPCS, acquired speckle patterns are processed via the *Dynamix* package (Paleo *et al.*, 2021 ▸) to obtain the intensity–intensity autocorrelation function pixel by pixel. This quantity is then averaged over a desired azimuthal range to derive the time (*t*) and ensemble averaged intensity–intensity autocorrelation function, *g*_2_(*q*, *t*). Further visualization and analysis of *g*_2_(*q*, *t*) data can be carried out using the *XPCSUtilities* program (Chèvremont, 2023 ▸).

## 3. Performance of SAXS and XPCS methods

This section describes selected SAXS and XPCS measurements, demonstrating the improvements compared with the previous-generation source. The full impact of fourth-generation synchrotron sources on these techniques is still being explored, and a better picture will emerge in the near future.

### 3.1. High-resolution USAXS

**Figure 1:**
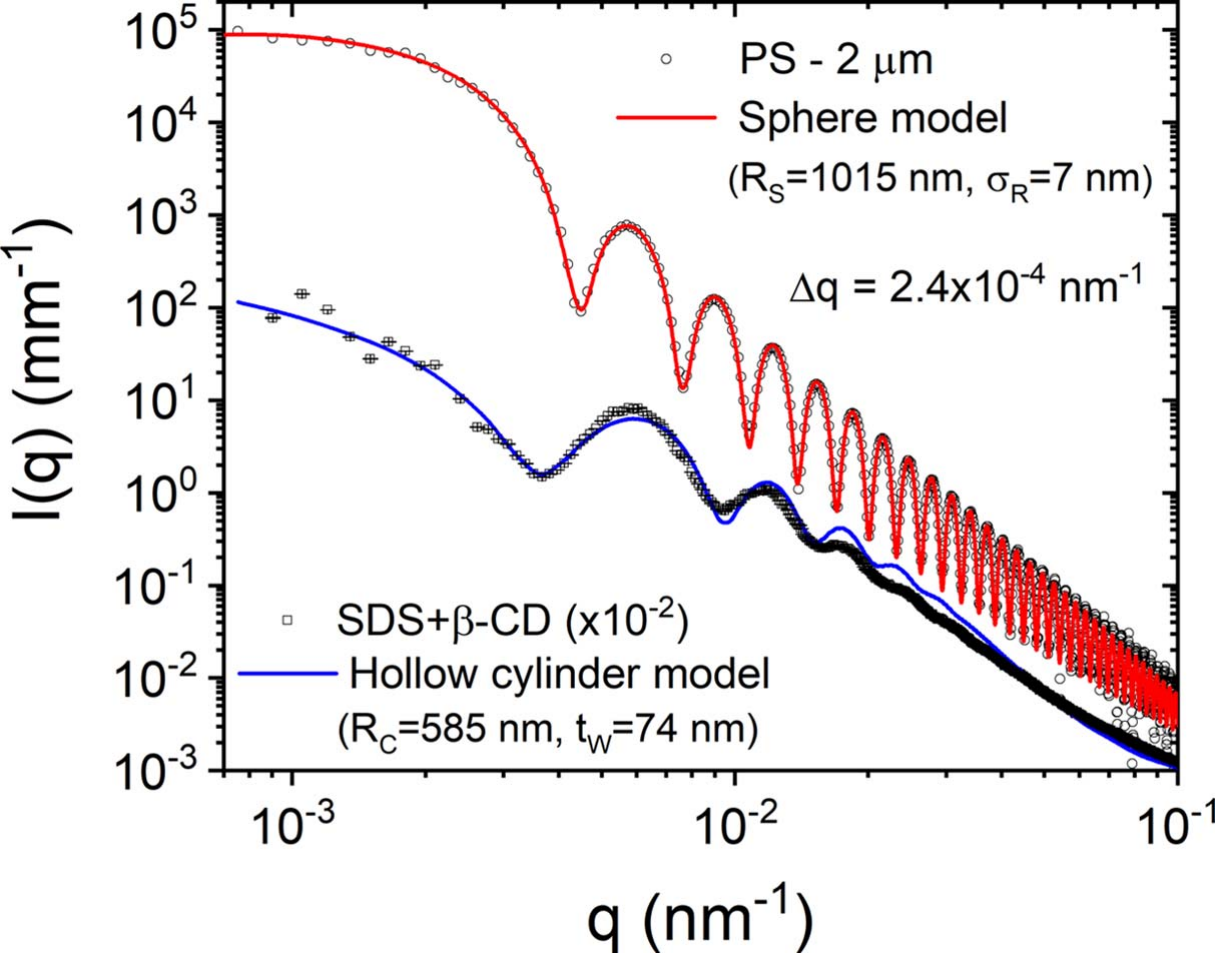
Background-subtracted scattering profiles from suspensions of dilute PS spherical colloids and self-assembled microtubes of SDS and β-CD in 1:2 molar ratio. The continuous curves are the corresponding models, polydisperse spheres and hollow cylinders scattering functions, with the size parameters indicated in the legend. For clarity, the lower profile has been shifted down by a factor of 100. These scattering profiles illustrate the high *q* resolution and detection capability that can be obtained.

A direct consequence of the reduced source emittance is the high degree of collimation that can be obtained with minimal optical components. This helps to preserve the spectral properties as well as to minimize the parasitic background. Traditionally, USAXS is performed using a Bonse–Hart instrument involving collimator and analyzer crystals (Ilavsky *et al.*, 2018 ▸; Pauw *et al.*, 2021 ▸). The smaller divergence and size of the beam enable USAXS to be performed in the pinhole collimation using a high-resolution 2D detector placed sufficiently farther from the sample (*ca* 30 m). This approach has many advantages, notably in time-resolved studies, and for weakly scattering and radiation-sensitive samples (Kishimoto *et al.*, 2014 ▸; Narayanan *et al.*, 2018 ▸). In addition, oriented specimens often require 2D detection for a quantitative analysis. Fig. 1 ▸ illustrates the high resolution that can be obtained and the ability to detect relatively weak scattering features very close to the primary beam over the USAXS range. These measurements were performed at a sample-to-detector distance of 31 m using λ ≃ 1.01 Å, and the FWHM *q* resolution (Δ*q*) of the setup was about 2.4 × 10^−4^ nm^−1^.

The first sample is a dilute suspension (volume fraction ∼ 0.01) of polystyrene (PS) microspheres in a mixture of water and ethanol (1:1 by volume). The normalized background scattering was accurately subtracted and, in addition, the secondary scattering contribution was corrected by two measurements with small and large beamstops (Narayanan *et al.*, 2022 ▸). The fine features of the scattering profile, *I*(*q*), correspond to the form factor of uniform spheres of mean radius *R*_S_ ≃ 1.015 µm and standard deviation (σ_*R*_) ≃ 7 nm (polydispersity ≃ 0.007). The second sample is an aqueous mixture (0.07 weight fraction) of surfactant sodium dodecyl sulfate (SDS) and polysaccharide β-cyclodextrin (β-CD) in a 1:2 molar ratio, which forms relatively long microtubes at room temperature (Ouhajji *et al.*, 2017 ▸; Landman *et al.*, 2018 ▸). The model curve is an approximate description by the form factor of long hollow cylinders with mean radius *R*_C_ ≃ 585 nm, wall thickness *t*_W_ ≃ 74 nm, and radius and thickness polydispersities of 0.08 and 0.6, respectively. These microtubes have a multilamellar hierarchical architecture, which can be seen at higher *q* values (Ouhajji *et al.*, 2017 ▸). The small Δ*q* and accurate subtraction of the parasitic background are critical for modeling of these scattering profiles.

**Figure 2:**
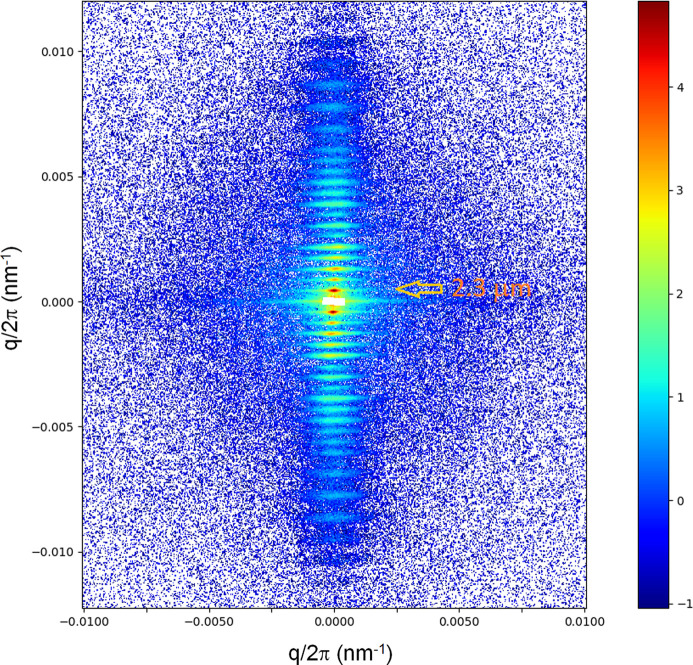
An ultra-low-angle diffraction pattern of a demebranated rabbit psoas muscle recorded with an X-ray exposure of only 5 × 10^9^ photons. The fiber axis is along the vertical direction and the well defined reflections correspond to the axial repeat of the sarcomeres of length ≃ 2.3 µm. Once again, the pattern demonstrates the high angular resolution and detection capability that can be achieved. The specimen is courtesy of M. Linari *et al.* (University of Florence, Italy).

The high resolution is useful for the investigation of larger-scale coherent structures and their structural dynamics. Fig. 2 ▸ displays the ultra-low-angle diffraction pattern from a mammalian rabbit skeletal muscle, which shows the axial repeat of sarcomeres (the unit cell of muscle). The first-order peak corresponds to a sarcomere length of ∼2.3 µm. The higher orders are modulated by the form factor (an interference function), which is determined by the mass distribution within the sarcomere. The instrument background has been subtracted from the measured pattern and, in addition, the gaps between the Eiger2 4M modules have been patched using the mirror symmetry of the diffraction diagram along the meridian and equator. The spacing and intensities of these reflections change upon activation of the muscle, which can be used to probe the structural dynamics of muscle regulation (Reconditi *et al.*, 2017 ▸; Brunello *et al.*, 2020 ▸).

### 3.2. Ultra-small-angle XPCS

The order of magnitude increase in the degree of coherence of the fourth-generation sources can be exploited for a variety of applications such as XPCS, coherent diffractive imaging *etc*. (Narayanan & Konovalov, 2020 ▸; Lehmkühler *et al.*, 2021 ▸). For XPCS, this aids both in terms of the speckle contrast (β) and the fastest dynamics that can be probed with an appropriate detector (Sinha *et al.*, 2014 ▸). This has already allowed probing of the dynamics of dense protein solutions (Chushkin *et al.*, 2022 ▸) and packed microemulsion droplets (Matthews & Narayanan, 2022 ▸).

**Figure 3:**
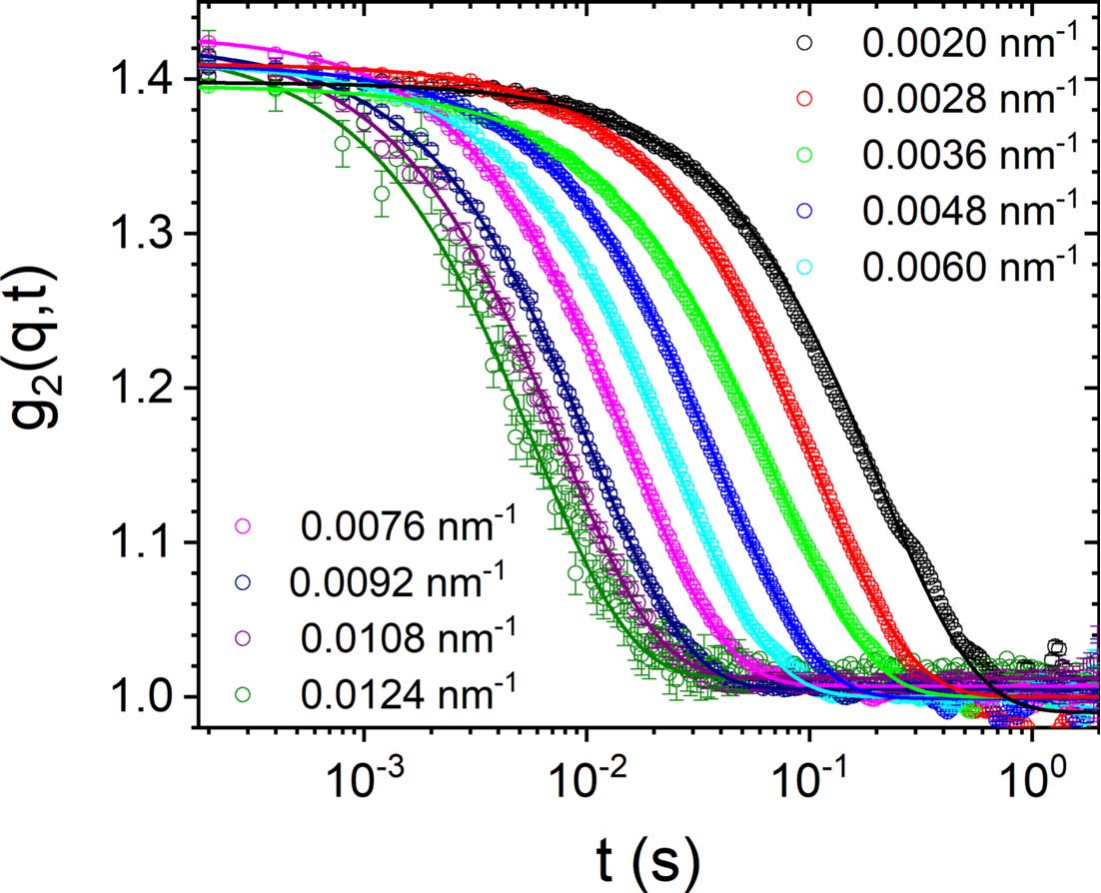
The time and ensemble averaged intensity–intensity autocorrelation functions [*g*_2_(*q*, *t*)] from a dilute colloidal suspension of silica particles with mean radius 300 nm. Continuous lines are exponential fits using equation (1) with *D*_0_ ≃ 0.58 µm^2^ s^−1^.

Fig. 3 ▸ depicts a typical time and ensemble averaged *g*_2_(*q*, *t*) as a function of *q* from a dilute silica colloidal suspension (volume fraction ∼ 0.01) with *R*_*S*_ ≃ 300 nm and polydispersity ≃ 1.8%. In this case, the sample scatters relatively high and therefore the beam intensity was attenuated by a factor of 30. Measurements were performed using the Eiger 500 K detector operating in the 8-bit mode, and 10 000 frames were acquired in 2 s. In this case, all *g*_2_(*q*, *t*) functions decay by an exponential function, as expected for Brownian particles. The measured *g*_2_(*q*, *t*) is related to the intermediate scattering function *g*_1_(*q*, *t*) via the Siegert relation, 

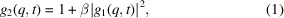

where β is determined by the coherence properties of the X-ray beam and the angular resolution of the setup (Sinha *et al.*, 2014 ▸), and increased from 0.3 to about 0.4 with the EBS (Zinn *et al.*, 2018 ▸). For Brownian particles, 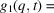 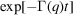 , where the relaxation rate Γ(*q*) = *D*_0_ *q* ^2^. *D*_0_ is the diffusion coefficient given by the Stokes–Einstein relation: *D*_0_ = *k*_B_ *T*/(6πη*R*_H_), where *k*_B_, *T*, η and *R*_H_ are the Boltzmann constant, absolute temperature, solvent viscosity and mean hydrodynamic radius of particles, respectively. The result in Fig. 3 ▸ demonstrates that multispeckle XPCS can be used to probe faster dynamics than purely diffusive motions. This aspect is illustrated in the case of directed motions either by diffusiophoresis (Gibbs, 2020 ▸) or subjected to a shear flow (Narayanan *et al.*, 2020 ▸). These systems display fast out-of-equilibrium dynamics, and multispeckle XPCS yields the ensemble averaged information in the bulk without any influence from the substrate.

### 3.3. XPCS studies of driven systems

The term ‘driven systems’ here implies that advection is more important than diffusion or, in other words, the dimensionless Peclet number 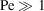 . Examples include self-propelled particulate suspensions, where each particle converts energy from the medium to perform directed motion (Singh *et al.*, 2017 ▸; Vutukuri *et al.*, 2020 ▸), or a suspension driven by an external field such as shear (Fuller *et al.*, 1980 ▸). In these cases, the propulsion or flow effects dominate over the Brownian diffusion and *g*_1_(*q*, *t*) can be factorized in the following form (Busch *et al.*, 2008 ▸; Burghardt *et al.*, 2012 ▸), 

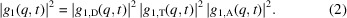

Here, the first term represents diffusive motions, the second term accounts for the transit effect of particles across the beam related to their mean velocity, *v*, and the last term is an advective term due to differences in the Doppler shifts of all particle pairs in the scattering volume, which is determined by the average velocity differences between all particle pairs, δ*v* (Fuller *et al.*, 1980 ▸). The exact functional form of *g*_1,A_(*q*, *t*) depends on the distribution of *v*. For a Gaussian distribution of δ*v* equation (2) can be approximated as (Zinn *et al.*, 2020 ▸) 

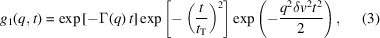

where *t*_T_ is the transit time of particles across the X-ray beam given by *t*_T_ = σ_B_/*v*, with σ_B_ being the Gaussian width of the beam.

A convenient method to realize self-propelled motion is by using Janus particles with a catalytic subunit, which when suspended in a catalytic medium such as hydrogen peroxide (H_2_O_2_) solution display autonomous motion induced by the chemical gradient around each particle (Ebbens & Howse, 2010 ▸). Using Janus particles composed of silica spheres with a hemispherical titania (anatase) cap suspended in H_2_O_2_ solution, this self-phoretic motion can be induced by illumination of ultraviolet (UV) light (Singh *et al.*, 2017 ▸; Vutukuri *et al.*, 2020 ▸). The magnitude of the mean propulsion velocity, *v*, depends on both the UV power and the concentration of H_2_O_2_ (fuel) (Zinn *et al.*, 2022 ▸).

**Figure 4:**
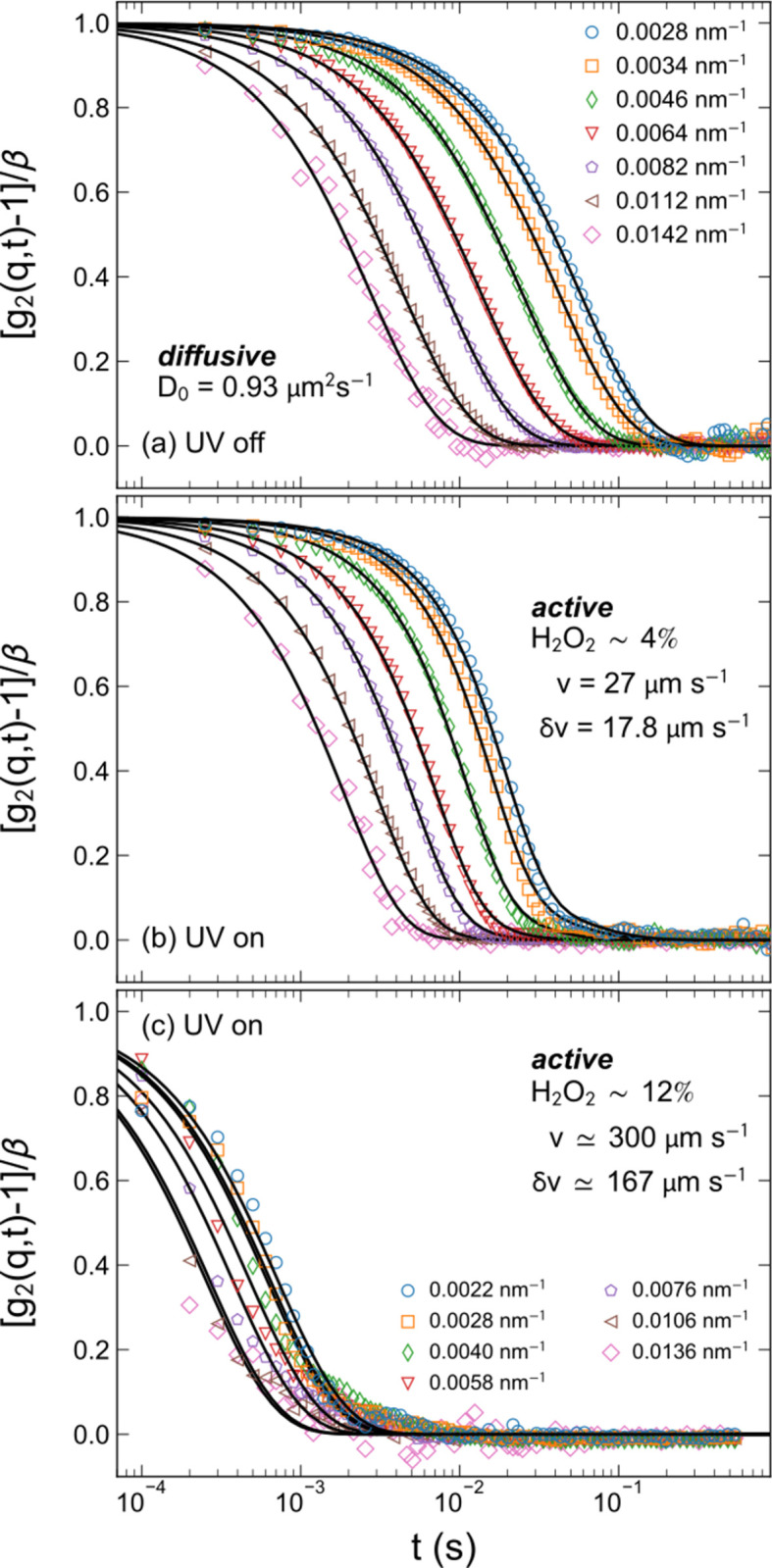
Typical ensemble averaged *g*_2_(*q*, *t*) functions at different *q* values (*a*) before and (*b*) after turning on the UV light for a sample containing 4% H_2_O_2_, and (*c*) for 12% H_2_O_2_ with the UV light on. The continuous lines are fitted curves using (*a*) equation (1) with the exponential term, and (*b*) and (*c*) equation (1) with equation (3). The main parameters are indicated in the legend. Adapted from Zinn *et al.* (2022 ▸).

Fig. 4 ▸ presents the steady-state dynamics of silica–titania Janus particles (*R*_S_ ≃ 260 nm and volume fraction ≃ 0.0003) in H_2_O_2_ solution with two different concentrations before and after turning on the UV light (Zinn *et al.*, 2022 ▸). In the absence of UV illumination, Fig. 4 ▸(*a*), particles behave as purely Brownian (passive) with an exponential decay of *g*_2_(*q*, *t*), as in Fig. 3 ▸. Upon UV illumination (nominal power 200 mW), the dynamics become much faster (active) and the corresponding *g*_1_(*q*, *t*) functions are described by equation (3), as shown in Figs. 4 ▸(*b*) and 4 ▸(*c*). The decay is dominated by the two Gaussian terms and the *q* dependence becomes weaker, as indicated by the compression of the curves into a narrower time range. The analysis enabled the deduction of *v* and δ*v* of the ensemble of particles in the scattering volume. Further increase of H_2_O_2_ for the same UV power displayed even faster dynamics, Fig. 4 ▸(*c*), and a weaker *q* dependence, as shown by the squeezing of *g*_2_(*q*, *t*) to an even narrower time range. Moreover, the functional form of *g*_1,A_(*q*, *t*) changed to an exponential, which would correspond to a Lorentzian distribution of *v*, as in turbulent fluids (Zinn *et al.*, 2022 ▸).

The above example demonstrates that multispeckle XPCS can be used to probe the emergence of fast active dynamics in dilute colloidal suspensions and derive the mean velocity, velocity fluctuations, and effective diffusion coefficient in three dimensions. This approach can be extended to a variety of active systems for deriving the statistical properties of the emergent dynamics. In this case, the dynamics changed from purely diffusive [Fig. 4 ▸(*a*)] to active [Fig. 4 ▸(*b*)] and then analogous to active turbulence [Fig. 4 ▸(*c*)], while the static scattering profile (form factor) remained unaltered (Zinn *et al.*, 2022 ▸).

**Figure 5:**
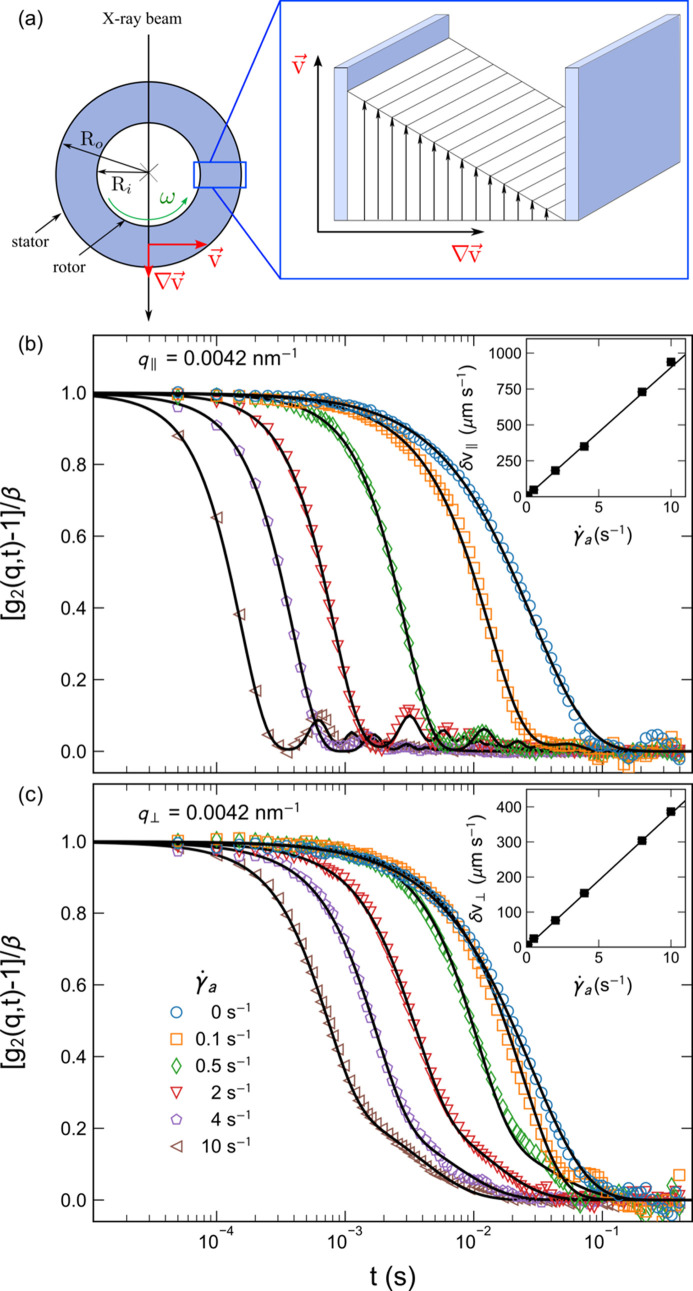
(*a*) A schematic representation of the shear geometry employed for rheo-XPCS with the inner cylinder rotating at an angular velocity, ω. Below this are shown representative *g*_2_(*q*, *t*) functions for a dilute suspension of silica particles (mean radius ≃ 300 nm) for different shear rates at *q* = 4.2 × 10^−3^ nm^−1^ along the (*b*) horizontal and (*c*) vertical directions in radial configuration. The continuous lines are fits to (*b*) equation (4) and (*c*) equation (1) with equation (3), with β ≃ 0.35. The undulations in *g*_2_(*q*, *t*) signify the dominance of the sinc term in equation (4). The insets present the deduced δ*v* as a function of 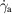 .

A second example is the case of suspensions subjected to a laminar shear flow in a Couette-type cell, which in this case consists of two concentric capillaries with the inner one coupled to a rheometer shaft (Narayanan *et al.*, 2020 ▸). Fig. 5 ▸(*a*) schematically depicts the shear geometry with inner and outer capillaries of radii *R*_i_ and *R*_o_, respectively, which in this case were 0.5 and 1 mm, respectively. The sample was contained in the annular space. These rheo-XPCS measurements were carried out using dilute silica colloids (*R*_S_ ≃ 300 nm and volume fraction ≃ 0.01) in water. In the ideal case of Couette flow, δ*v* is constant across the gap and essentially zero in the two transverse directions (Burghardt *et al.*, 2012 ▸). In this case, *g*_2_(*q*, *t*) along the flow direction can be expressed in the following form, 

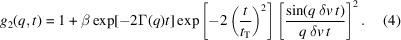

In the radial direction, the scattering volume is twice the annular gap and the decay of *g*_2_(*q*, *t*) is dominated by the largest δ*v* ≃ *v*. Figs. 5 ▸(*b*) and 5 ▸(*c*) display representative sector averaged (azimuthal range ±10°) *g*_2_(*q*, *t*) functions in the radial configuration (*i.e.* the X-ray beam passing through the shear gradient) along the horizontal and vertical directions, respectively, for different apparent shear rates ( 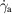 ). The data analysis was performed by simultaneous fits over 2.8 × 10^−3^ ≤ *q* ≤ 1.2 × 10^−2^ nm^−1^. Even at a very low 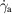 (≤0.1 s^−1^), a deviation from purely diffusive behavior is evident. In the horizontal direction, the shear planes are well defined, as indicated by the clear oscillations arising from the advective term in equation (4). The influence of the transit term was not significant in the analysis. The magnitude of δ*v* in the vertical direction is convoluted by different contributions, including the transverse variation of *v* across the gap. In the ideal case, δ*v* ≃ 0 in the vertical direction. Moreover, the decay of *g*_1_(*q*, *t*) was found to be Gaussian, as in equation (3). The insets show the variation of deduced values of δ*v* as a function of 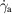 . For this shear geometry, 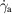 set by the rheometer control software is larger than the real shear rate, 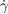 , by a factor of 1.67 (Narayanan *et al.*, 2020 ▸). As expected, δ*v* follows a linear relationship with 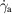 and XPCS yields the local shear rate. In the radial configuration, along with the linear increase of δ*v*, the value of the diffusion coefficient also increased linearly from *D*_0_ ≃ 0.9 µm^2^ s^−1^ to about 12.3 µm^2^ s^−1^ for 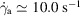 .

The XPCS results presented here demonstrate that it is possible to decouple the change in the intrinsic dynamics of the sample from the Doppler shifts caused by the shear flow (velocity differences) in the measured *g*_2_(*q*, *t*), as also shown by earlier studies (Busch *et al.*, 2008 ▸; Burghardt *et al.*, 2012 ▸). The statistical properties of low Reynolds number flows can be probed by this method. In particular, the inhomogeneities in the flow can be monitored at small size scales that are not accessible for the particle imaging velocimetry technique. At present, the measurement range is limited to about 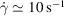 due to the frame rate of the detector (23 kHz), but with the availability of even faster detectors, measurements can be extended to larger shear rates.

### 3.4. Radiation damage

The increased brilliance and detection capability also reveal the limitations of the technique, which may hamper reaching the expected performance. Radiation damage is the most serious issue for the vast majority of soft matter and biological specimens, which needs to be identified and rectified at the early stage of any study (Jeffries *et al.*, 2015 ▸; Narayanan *et al.*, 2014 ▸). The onset of radiation damage depends not only on the X-ray dose but also on the prevailing physicochemical conditions. Since most samples have to be investigated under specific thermodynamic or physiological conditions, appropriate protocols need to be adopted for each system. The threshold of damage can be assessed by progressively increasing the exposure time (from a few milliseconds) and the period in between successive exposures. The onset of damage is judged on the basis of a systematic change in the scattering profile with increasing X-ray exposure. Typically, in a SAXS setup, the focusing and collimation are adjusted such that the beam size is minimum on the detector and relatively large on the sample. This condition cannot be met when a small beam spot on the sample or a coherent beam is required. In that case, the flux, exposure time and delay between exposures need to be optimized to remain below the damage threshold.

**Figure 6:**
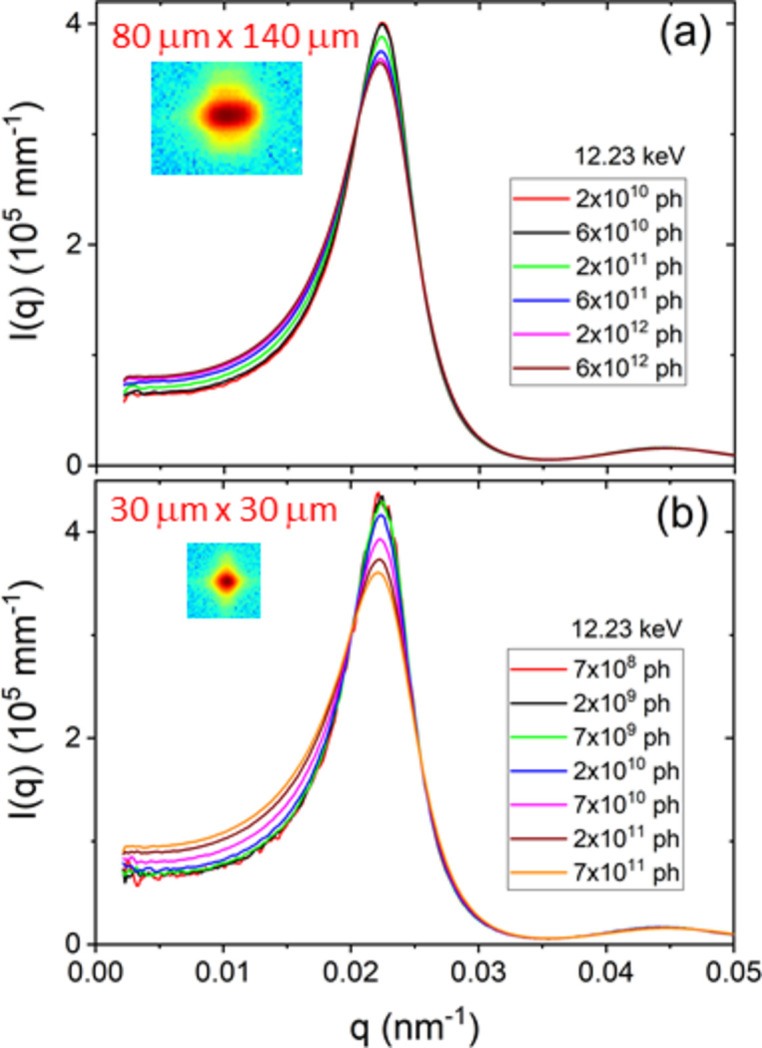
X-ray beam induced changes on the effective structure factor, *S*_M_(*q*), of a concentrated silica particle suspension for two different beam sizes corresponding to (*a*) SAXS and (*b*) XPCS configurations. With a larger beam (*a*), the onset of the irradiation effect occurs at a higher intensity of the incident beam, but in both cases the threshold is at a comparable photon flux value.

Fig. 6 ▸ displays the effect of radiation dose on the effective structure factor [*S*_M_(*q*)] peak of a concentrated suspension of charge-stabilized silica colloids (*R*_S_ ≃ 126 nm and volume fraction ≃ 0.43) for two different beam sizes. To facilitate the comparison of the *S*_M_(*q*) peak and the compressibility limit [*S*_M_(*q* ≃ 0)], data are presented with *I*(*q*) on a linear scale. The larger [Fig. 6 ▸(*a*)] and smaller [Fig. 6 ▸(*b*)] beams correspond to standard SAXS and XPCS configurations, respectively. In this case, the sample was made sensitive to radiation by tuning the volume fraction close to the colloid freezing transition. If left unperturbed the sample would crystallize and Bragg peaks of colloidal crystals would appear with time. As the X-ray exposure is systematically increased, the *S*_M_(*q*) peak and the compressibility limit, *S*_M_(*q* ≃ 0), manifest significant changes analogous to an increase in the ionic strength of the suspension (Westermeier *et al.*, 2012 ▸). With the smaller beam, the apparent onset of radiation damage is an order of magnitude earlier in terms of number of photons but roughly corresponds to the same photon flux (*i.e.* number of photons normalized to the beam cross section) as with the larger beam. Moreover, in the case of the smaller beam, the radiation-induced changes persist without leveling off.

## 4. Summary and outlook

The previous section has illustrated some representative advances in the USAXS and UA-XPCS methods at the ID02 beamline with the advent of the fourth-generation EBS. However, the examples are not exhaustive as the new generation sources are only beginning to be exploited. For a given undulator and electron energy, third- and fourth-generation sources deliver comparable monochromatic beam intensity, as measured by the number of photons per second. The brightness is increased primarily due to the reduction in the beam size and divergence in the horizontal direction. As a result, conventional static SAXS and time-resolved SAXS measurements may not significantly benefit from the new source properties compared with the gain due to advanced detectors. The differences emerge when high angular resolution and larger coherence length are required, such as for USAXS in the pinhole collimation, or a smaller beam spot is needed, such as for scanning SAXS and WAXS.

With the advent of fourth-generation sources and availability of advanced pixel array detectors, spatial and temporal scales accessible for USAXS/SAXS/WAXS techniques have significantly broadened (Narayanan *et al.*, 2022 ▸). Similarly, XPCS can now be performed on dilute samples with reasonably good scattering contrast (Zinn *et al.*, 2022 ▸) and dense systems with relatively low contrast such as proteins (Chushkin *et al.*, 2022 ▸). The increase in the transverse coherence is essential for probing larger size scales by scattering methods (Shinohara & Amemiya, 2015 ▸). The high degree of coherence may become a nuisance in a conventional SAXS analysis as the speckles in the scattering pattern make the Guinier region and Bragg peaks somewhat noisy. To benefit from the beam coherence, the data analysis needs to be pushed beyond the conventional approaches, and in particular towards the statistical properties of scattering as developed in the light scattering domain (Scheffold & Cerbino, 2007 ▸).

Radiation damage is a major issue when exploiting extremely brilliant sources. The smaller beam size proportionally decreases the threshold for the onset of radiation damage in the case of synthetic soft materials and biological specimens. In such cases, a larger beam cross section becomes an advantage at the expense of angular or spatial resolution. A larger beam with a single coherence area is optimum for XPCS measurements on radiation-sensitive samples.
